# The impact of the moon cycles’ in different seasons on heart failure patients’ hospitalization and length of stay

**DOI:** 10.1097/MD.0000000000041614

**Published:** 2025-02-28

**Authors:** Bashar Khiatah, Sam Jazayeri, Brian Diep, Naofumi Yamamoto, Anna Belikova, Graal Diaz, Amanda Frugoli, Craig Mansour

**Affiliations:** a Department of Internal Medicine, Overlake Medical Center, Bellevue, WA; b Department of Research and Statistics, Community Memorial Hospital, Ventura, CA; c Department of Interventional Cardiology, Cardiology Associates Medical Group, Ventura, CA.

**Keywords:** gravitational pull, heart failure, lunar cycle, solstice

## Abstract

The natural forces of the lunar cycle and seasonal solstice variation on the water surfaces have been studied extensively but not on patients with fluid problems such as heart failure (HF). This retrospective review investigates these temporal effects on admission rates of patients with heart failure and length of stay. In this study, we try to answer the following questions: Do moon cycles (full moon vs new moon) significantly affect the number of patients admitted? and Do moon cycles significantly affect the patient’s hospital length of stay (LOS?). All patients with HF exacerbation admission between January 1, 2016 to December 31, 2019, were filtered according to admission date based on the lunar calendar. Patients admitted on the day of, the day before, and the day after a new and full moon were included. Question 1, Poisson regression models were employed. The overdispersion obtained from the AER package was 1.63. All analyses were performed using R (R Core Team). A total of 758 patients were admitted during lunar cycles, 50.1% (N = 380) were admitted during the new moon and 49.4% (N = 378) during the full moon. The mean age is 78.4 (SD 7.2), the mean BMI is 28.8 (SD 6.7), and the mean LOS is 5.6 (SD 3.4) with no significant differences in patients admitted during both of the moon cycles. The seasons variable showed statistically significant coefficients, with the summer season (S2) having the highest impact (coefficient 0.85, *P* = .001). Some interaction between Moon-Cycle, summer season, and BMI influenced patient admissions during lunar cycles (coefficient = 0.49 *P* ≤ .001). This study showed that the moon cycle may impact patients with HF during the summer season only. Prospective studies are needed on a national level to investigate further the impact of the moon cycle on HF patients. This will help improve patient outcomes and pathogenesis, and there is excellent potential for reducing medical costs.

## 1. Introduction

Heart failure is a clinical syndrome due to structural and functional abnormalities in the myocardium impacting the ventricular filling or the ejection of the blood.^[1]^ The entirety of the disease is the failure of blood volume circulation. Heart failure has a significant quality of life impact and effects on health care spending with approximately $30.7 billion spent on direct medical costs of heart failure (HF), with a projected increase of ≈127%, reaching $69.7 billion by 2030.^[2]^ Environmental factors are underappreciated risk factors contributing to the stability or development of heart failure exacerbations. Only a handful of studies have been performed to evaluate the effects of weather and air quality on this patient population.^[3]^ The role of the lunar cycle on heart failure exacerbations remains unknown.

There are hospital legends regarding the lunar cycle and seasons that affect hospital census for specific diseases. Some of these have been backed by evidence, including a seasonal variation for cardiac disease outside of atherosclerotic plaque rupture.^[4]^ In 1996 Ornato et al completed an extensive retrospective review using the National Registry of Myocardial Infarction.^[1]^ Over 83,541 patients were reviewed, and the study concluded there was a seasonal pattern to acute myocardial infarction and National Registry of Myocardial Infarction.^[4]^ This study design was repeated in 2017 by Nagarajan et al^[5]^ using the Get With The Guidelines-Coronary Artery Disease (GWTG-CAD) database. They were able to demonstrate that acute myocardial infarction (AMI) admissions varied across seasons (*P* < .01) and were higher in winter (winter vs spring n = 21,483 vs 20,291, respectively).

In addition to seasonal outcomes in acute myocardial infarction, studies have investigated the lunar cycles on cardiac surgery outcomes. These studies have mainly focused on cardiac surgery for aortic dissection repair, but the studies have had surprising outcomes. Shuhaiber et al^[6]^ examined the influence of seasons and lunar cycle on hospital outcomes following ascending aortic dissection repair from January 1996 to December 2011. They identified that season did not affect mortality or length of stay. They did discover that the lunar cycles did have an effect, with a full moon reducing the odds of death and shorter length of stay. This study was repeated to include 2 continents by Ma et al,^[7]^ who examined acute aortic dissection’s chronologic and climatic factors. They uncovered that acute aortic dissection exhibits significant chronologic variation with a peak in winter and a possible correlation between mean temperature and atmospheric pressure.^[8]^ Another study, evaluating mood and exercise performance with the lunar cycle and time of day, identified that short-term maximal performance was impacted by the new moon and morning time of day.^[9]^

**Figure 1.**
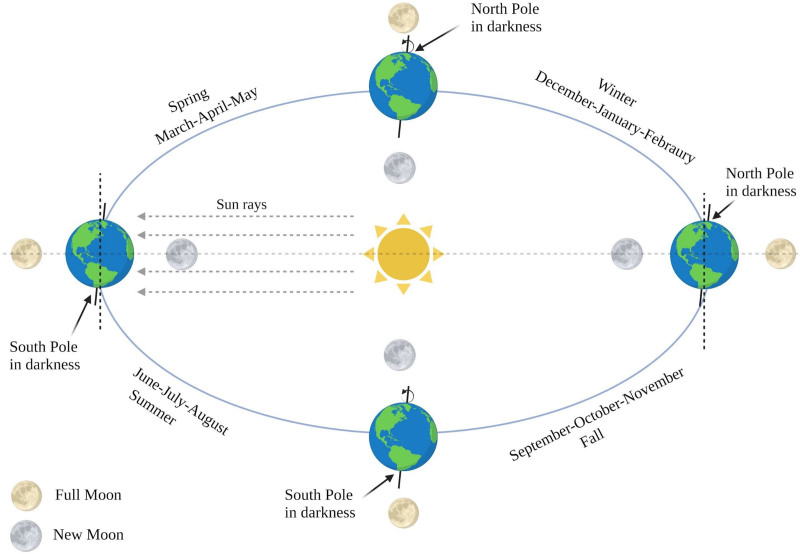
Earth’s axis during different equinoxes and lunar cycle.

Science has revealed that the tides are a direct effect of the gravitation pull of the moon on these bodies of water. They have also demonstrated that the tides are affected most during the lunar cycles. We hypothesize that disease states associated with hypervolemia will be affected by the lunar cycles as well. In this retrospective research design, we target the most prevalent fluid-overloaded state- heart failure and investigate if heart failure admissions are affected by the lunar cycles (Fig. 1).

### 1.1. Research questions

1. Do moon cycles (full moon vs new moon) in different seasons significantly affect the number of patients admitted?
2. Do moon cycles significantly affect the patient’s length of stay (LOS)?

## 2. Materials and methods

This is a retrospective analysis of patients with heart failure exacerbation requiring hospital admission to our institution (Community Memorial Hospital) from January 1, 2016, to December 31, 2019. These dates were chosen due to limited data availability before January 2016. Ethical approval was granted by the IRB committee of Community Memorial Hospital and patient consent was waived due to the study nature. All patients aged 18 and above who were admitted with an ICD code including acute congestive heart failure (CHF) exacerbation acute on chronic CHF exacerbation, acute heart failure preserved ejection fraction, acute heart failure reduced ejection fraction, shortness of breath on exertion, shortness of breath, acute respiratory failure, lower extremity edema, anasarca, chest pain, hypoxia, and fatigue, were used. Patients’ demographics (age, gender and BMI), presence of diabetes, hypertension and hyperlipidemia, date of admission, length of stay, BNP values on admission and ejection fraction. All patients were anonymized. Afterward, all patients’ charts were reviewed and narrowed down further after accounting for the Lunar cycle based on national oceanic and atmospheric administration.^[9]^ All patients admitted on the day of, the day before, and the day after a new and full moon were included in the analysis. Thereafter, the patient has been divided into 4 different seasons based on meteorological seasons criteria. This includes winter (which starts in December through February), spring (which starts in March through May), summer (June through August) and fall (September through November).^[9]^

The data was verified that each patient met the criteria for CHF exacerbation including symptoms, radiographic diagnostic imaging, brain natriuretic peptide (BNP) results, echo results, and physical exam reported in history and physical examination of illness notes on admission. All the data was later on divided into different solstice (summer and winter) and seasons. The 29-day lunar calendar month was divided into 4 phases of a new moon, waxing moon, full moon, and waning moon. Each phase was apportioned as follows: Day 1–7 = new moon, 8–14 = waxing moon, 15–21 = full-moon, and 22–29 = waning moon. This was also divided into separate summer and winter solstice as the moon’s distance from the Earth is different and certainly affects gravitational force.

Each season was divided into 2 moon phases as mentioned previously (full moon and new moon phases), and the mean number of patients and average LOS in each moon phase were employed as outcome variables. Patient’s age, gender, body mass index (BMI), and whether or not they had preserved ejection fraction (PEF), diabetes mellitus (DM), and arrhythmias (ARR) were aggregated per each moon phase within a season for utilization as covariates (e.g., the proportion of males during the full moon in spring).

### 2.1. Statistical analysis

Descriptive and bivariate statistics were completed and summarized in (Table 1). To investigate Research question 1, Poisson regression models were employed. The overdispersion obtained from the AER package^[10]^ was 1.63. Thus, no statistical adjustment was required.^[11]^ For Research question 2, gamma regression models with the log link were employed to fit LOS data. Among various approaches to fit LOS data, including the use of lognormal and Weibull distributions, a gamma regression approach was utilized due to the robustness to non-heavy tail shapes with high error variance^[12]^ and its modeling flexibility. For comparison purposes, winter and the new moon phases were used as reference groups. All analyses were performed using R (R Core Team, 2021).

**Table 1:** Data consortium: patients admitted for heart failure exacerbation for the period January 1, 2016 to December 31, 2019.

	N	Percent (%)
Total admitted CHF patients	6083	
Total admitted CHF patients during the lunar cycle	758	12.5
New moon	380	50.1
Summer solstice	195	
Winter solstice	185	
Full moon	378	49.9
Summer solstice	190	
Winter solstice	188	

CHF = congestive heart failure.

## 3. Results

A total of 6083 patients were admitted for heart failure exacerbation for the study period. Of these patients, 12.5% (N = 758) were admitted during lunar cycles, of which 50.1% (N = 380) were admitted during the new moon and 49.4% (N = 378) during the full moon (Table 1). Among patients admitted during lunar cycles, the mean age is 78.4 (7.2), the mean BMI is 28.8 (6.7), and the mean LOS is 5.6 (3.4). Variance analysis showed no significant differences in patients admitted by season on mean age, BMI, and LOS (Table 2). A generalized linear model (GLM) analysis suggests that Moon-Cycle did not significantly impact the number of patients (coefficient = 0.25, *P* = .08; Table 3). The season variable shows statistically significant coefficients, with the summer season (S2) having the highest impact (coefficient 0.85, *P* = .001). It implies that S2 has a strong positive association with the number of patient admissions during lunar cycles. Some interaction terms involving Moon-Cycle and BMI are present in the model. For example, “Moon cycle: S2: BMI” has a highly significant negative coefficient (coefficient = 0.49 *P* ≤ .001), suggesting that the interaction between Moon-Cycle, S2, and BMI strongly influences patient admissions during lunar cycles (Table 4).

**Table 2:** Descriptive analysis of admitted CHF patients during lunar cycles by season (N = 758).

Variable	Full	New	*P* value
Mean age			
Fall	78.3	78.4	NS
Winter	77.3	80.0	
Spring	78.3	78.9	
Summer	77.5	78.2	
Mean BMI			
Fall	29.7	30.4	NS
Winter	28.4	28.6	
Spring	28.6	28.5	
Summer	28.9	27.4	
Mean length of stay			
Fall	5.2	5.4	
Winter	6.4	5.7	
Spring	5.8	5.6	NS
Summer	4.9	5.8	

Analysis of variance showed no significant differences by season in mean age, BMI, or LOS.

The significance level was set at < 0.05.

BMI = body mass index, CHF = congestive heart failure, LOS = length of stay.

**Table 3:** Generalized linear model analysis using Poisson family distribution to indicate the relationship between patient admissions and predictor variables.

Variable	Coefficient	Standard error	*P* value
Moon cycle	0.25	0.15	.09
S1	0.19	0.15	.2
S2	0.85	0.24	<.001***
S3	0.33	0.34	.34
c_M_BMI	0.15	0.07	.03*
Moon cycle: S1	−0.12	0.2	.54
Moon cycle: S2	−1.25	0.29	<.001***
Moon cycle: S3	−0.47	0.39	.21
S1: c_M_BMI	−0.03	0.09	.77
S2: c_M_BMI	0.49	0.18	.01**
S3: c_M_BMI	−0.25	0.21	.22

BMI = body mass index, S1 = spring, S2 = summer, S3 = fall, S4 = winter.

* *P* ≤ .05.

** *P* ≤ .01.

*** *P* ≤ .001.

**Table 4:** Generalized linear model analysis for length of stay.

Variable	Coefficient	Standard error	*P* value
Intercept	1.72	0.1	.001**
c_M_BMI	0.09	0.03	.01*
Moon cycle: S1	−0.15	0.2	.46
Moon cycle: S2	−0.47	0.21	.03
Moon cycle: S3	0.21	−0.72	.48

BMI = body mass index, S1 = spring, S2 = summer, S3 = fall, S4 = winter.

* *P* ≤ .05.

** *P* ≤ .001.

## 4. Discussion

CHF is, by definition, a fluid problem. The mainstay treatment for acute CHF exacerbation includes diuretics, fluid restriction, and sodium restriction, directly affecting fluid status. The lunar cycle is known to create low and high tides on Earth’s vast ocean surfaces, but little is known about the significance of its gravitational effect on the human’s intravascular microenvironment. Although several different studies have elucidated an association with major cardiovascular diseases such as coronary artery disease and ascending aortic dissection, very few theoretical models and even less research exist on the influence of the full and new moon on heart failure exacerbation during certain seasons in a hospital emergency room setting.

This limited novel study design examines a diverse patient population undergoing HF exacerbation admission in a Southern California community hospital across 4 seasons subdivided by complete and new moon phases. The findings demonstrated that summer and BMI independently and combined increased the number of patients admitted with HF exacerbation. The BMI had a further impact with an increase in the length of stay for all of these patients. When considering the lunar cycle, there was a significant decrease in the patients’ number during the full moon in summer -despite its effect and the BMI impact on these patients. The length of stay was also significantly reduced during the same time. As previously mentioned, heart failure exacerbation is correlated with volume retention, it is possible that excess weight from fluid accumulation may impact BMI and result in a BMI above their baseline.

For this reason, weight is often used as a parameter for volume status. As expected, we found that elevated BMI was directly correlated with the number of hospital visits for CHF exacerbation. However, when we evaluated the number of visits during the full moon in the summer, we found a statistically significant reduction in the number of visits regardless of BMI. In contrast, a statistically significant increase in hospital visits was seen during the summer’s new moon phase. We hypothesize this is related to the difference in gravitation pull from the summer solstice and the natural tilt of Earth’s axis as this correlated to the neap tides. This could be the reason for the decreased length of stay, where diuretics work faster in achieving euvolemia, allowing for a faster discharge and decreasing the cost of treating these patients. In the evaluation of winter and the new moon, the impact is decreasing patient volume that nearly reaches a statistical significance, which is precisely the same moon’s position regarding the earth access as seen in Figure 1. The relation between the moon and the Earth’s axis at that time appears to favor patients with failing hearts. The seasonal variation may contribute to the pathophysiology of heart failure exacerbation. It is known that the Earth’s distance from the sun is at its furthest during the summer months, correlating with a minute reduction in gravitational pull. This proportionately increases the gravity that our moon exerts on the Earth. While this effect on the organismic level is unclear, it may have some role in manipulating our metabolic demands. It allows the body to compensate longer despite volume retention in heart failure. It should be recognized that the theoretical method behind these findings is uncertain.

There was no significant difference between males and females, heart failure type (preserved or reduced), the presence or arrhythmia, and diabetes in either LOS or the number of hospital admissions. More studies are needed at a global level to confirm these findings. If these findings are supported in repeat studies involving continents on each side of the equator, it is possible that patient-specific applications could be utilized to alert patients to change fluid intake or diuretic needs during the time of the solstice and lunar cycles as a method to prevent heart failure exacerbations. The ultimate confirmation of these results could be achieved using pulmonary artery pressure monitoring systems and correlation to the moon’s cycle and specific gravitational pull based on location.

## 5. Conclusion

This study has shown the potential impact of the moon cycle on patients with heart failure. Further research is needed to confirm our findings on a global scale. This information may be able to have a positive impact on patients with heart failure and could be integrated into the growing medical technologies to prevent heart failure exacerbation hospitalizations and overall financial burden.

## Author contributions

**Conceptualization:** Bashar Khiatah, Sam Jazayeri, Amanda Frugoli, Craig Mansour.

**Data curation:** Bashar Khiatah, Sam Jazayeri, Brian Diep, Naofumi Yamamoto, Anna Belikova.

**Formal analysis:** Bashar Khiatah, Brian Diep, Naofumi Yamamoto, Anna Belikova, Graal Diaz.

**Investigation:** Bashar Khiatah, Amanda Frugoli.

**Methodology:** Bashar Khiatah, Amanda Frugoli, Graal Diaz, Craig Mansour.

**Writing – original draft:** Bashar Khiatah, Amanda Frugoli, Graal Diaz.

**Writing – review & editing:** Bashar Khiatah.
